# Use of single-cell technology to improve our understanding of the role of TLR2 in macrophage–*Mycobacterium tuberculosis* interaction

**DOI:** 10.1128/msystems.00730-23

**Published:** 2023-10-03

**Authors:** Alexandre Gouzy

**Affiliations:** 1 Department of Microbiology and Immunology, Weill Cornell Medical College, New York, New York, USA; University of California San Diego, La Jolla, California, USA

**Keywords:** *Mycobacterium tuberculosis*, macrophage, TLR2, single-cell RNA sequencing

## Abstract

The interaction between *Mycobacterium tuberculosis*, the agent of tuberculosis (TB), and its host cell, the macrophage, is multifaceted, dynamic, and involves multiple molecular partners. A better understanding of this interaction could help researchers manipulate the immune system in order to design host-targeted immunotherapies and/or develop a novel vaccine protecting better adults against TB. Jani and coworkers studied the role of the macrophage receptor TLR2 in the response to *M. tuberculosis* using single-cell technologies (C. Jani, S. L. Solomon, J. M. Peters, and S. C. Pringle, et al., mSystems, https://doi.org/10.1128/msystems.00052-23, 2023). This work addresses the multiple challenges associated with such studies and shows how informative single-cell analysis can be for the study of heterogeneous and complex host-pathogen interactions.

## COMMENTARY

Pathogen recognition is a key component of innate immunity, which informs of the nature of the pathogen encountered and allows its early clearance. Pathogen recognition is also an essential step toward the mounting of a more specific host response, the so-called adaptive immune response, that is necessary for eliminating recalcitrant pathogens (1, 2).

Host cells recognize bacterial pathogens with pattern-recognition receptors (PRRs) of different nature and localization. Among PRRs, Toll-like receptors (TLRs) were the first identified and are the best characterized so far (3). TLRs are transmembrane proteins localized either at the cell surface or within intracellular vesicles that recognize pathogen-associated molecular patterns (PAMPs), either secreted or present at the surface of the microbes. TLRs allow the recognition of a wide variety of PAMPs in the form of nucleic acids, proteins, and lipids (2, 4, 5). PAMPs are often invariable structures whose expression is vital to the bacteria, such as the lipopolysaccharide for Gram-negative bacteria, teichoic acid for Gram-positive bacteria, or the widespread peptidoglycan polymer (4).

*Mycobacterium tuberculosis* (Mtb), the etiologic agent of tuberculosis (TB), is a pathogen extensively studied for its interaction with its human host as being the first human killer among bacterial pathogens (WHO, Global TB Report 2022). TB affects mainly the lungs, where the bacteria interact with a variety of immune and non-immune cells. Among those cells, the study of macrophages is taking the most attention, as this cell type is the primary site of Mtb recognition, replication, and containment (6 – 8).

Mtb PAMPs are recognized by macrophages with TLRs such as TLR4 and TLR2 (9, 10). Jani et al. compared the early transcriptional response of macrophages to Mtb PAMPs and live Mtb and focused on the role of TLR2 in this process (11). PAMPs tested were lipids of various nature, including pthiocerol dimycocerosate, trehalose dimycolate, sulfolipid-1, mannose-capped-lipoarabinomannan, and phosphatidylinositol mannoside 6 (PIM6), as well as a total cell wall extract of Mtb. This study confirmed that Mtb elicits mainly two types of responses in macrophages, namely the NF-κB and the type I IFN-dependent signaling pathways. The authors found that the type I IFN response is mainly activated in response to live Mtb. This observation agrees with previous work showing that the induction of the type I IFN response in macrophages requires the presence of live mycobacteria expressing an intact type VII secretion system (ESX-1) (12 – 15).

On the other hand, the NF-κB response was stimulated by both live Mtb and PAMPs. Interestingly, the NF-κB response was weaker for live and dead Mtb in comparison to isolated PAMPs. The authors conclude that this difference in NF-κB induction levels between whole Mtb bacilli and isolated PAMPs might rely on the differences in antigen recognition of isolated PAMPs versus PAMPs included in the Mtb cell wall. In addition to this difference in antigen recognition, it is possible that PAMPs present at the surface of Mtb dampen the NF-κB response triggered by other PAMPs, as previously demonstrated for sulfoglycolipids antagonizing Mtb PAMPs recognition by TLR2 (16). Interestingly, Mtb-infected macrophages and bystander macrophages (non-infected macrophages) show overall a similar transcriptional response, reflecting some level of communication between infected and non-infected cells and/or the activity of Mtb-secreted PAMPs and/or PAMPs-containing exosomes on noninfected cells (17).

The authors show that in murine macrophages, the NF-κB response to PIM6 is uniquely dependent upon TLR2 and that PIM6/TLR2 interaction makes a unique contribution to the macrophage response to Mtb. The authors also show that the earliest TLR2-dependent transcriptional responses observed in *in vitro* cultivated macrophages are conserved *in vivo* and that a lack of TLR2 in mice impacts the quantity and phenotype of cellular subsets that emerge post-infection.

In apparent contradiction with the important role of TLR2 in Mtb recognition, the authors show that mice lacking TLR2 are as susceptible to TB as wild-type mice at 6 wk post-infection. This observation agrees with previous studies and emphasizes the controversial role of TLR2 in TB protection in mice (18 – 21). Differences in the genetic background of the mice and the bacteria used, as well as of the infection protocol employed, could be the cause of the discrepant results observed regarding the role of TLR2 in the protection against TB in mice. Moreover, TLR2 activation itself was shown to have both a positive and a negative impact on the control of Mtb infection, which might add to the complexity of the interpretation of the results (9, 22 – 24).

Importantly, many studies have shown that TLR2 has a protective role against TB in humans, as specific polymorphisms in the *TLR2* gene are associated with a higher risk of developing TB (25 – 28). The dissimilar role of TLR2 in the protection against TB in human and mice could originate from the difference(s) in the nature of the TLR2-dependent response(s) in those organisms. For example, an antimicrobial TLR2-dependent and vitamin D-mediated innate immune response is present in humans but absent in mice (29).

Altogether, the authors highlight the complexity and heterogeneity of the macrophage response to Mtb infection. Other factors, such as the variability in the genetic background of the host and the bacteria (30), the origin, and status of the infected macrophages (31) as well as the phenotype of the ingested bacteria (32), might influence the outcome of such interactions and increase the complexity of their study. To tackle those challenges, this work, among others, advocates for the use of single-cell technologies, including single-cell RNA sequencing and Flow Fluorescent *In Situ* Hybridization, to allow in-depth and unbiased profiling of immune cell populations and study the fate of individual bacteria in isolated immune cells (33 – 35).
